# Characterization of the Porcine CLEC12A and Analysis of Its Expression on Blood Dendritic Cell Subsets

**DOI:** 10.3389/fimmu.2020.00863

**Published:** 2020-05-13

**Authors:** Belén Álvarez, Elvira Nieto-Pelegrín, Paloma Martínez de la Riva, Daisuke Toki, Teresa Poderoso, Concepción Revilla, Hirohide Uenishi, Angel Ezquerra, Javier Domínguez

**Affiliations:** ^1^Departamento de Biotecnología, Instituto Nacional de Investigación y Tecnología Agraria y Alimentaria (INIA), Madrid, Spain; ^2^Animal Research Division, Institute of Japan Association for Techno-Innovation in Agriculture, Forestry and Fisheries, Tsukuba, Japan; ^3^Animal Bioregulation Unit, Division of Animal Sciences, Institute of Agrobiological Sciences, National Agriculture and Food Research Organization (NARO), Tsukuba, Japan

**Keywords:** CLEC12A, C-type lectin, dendritic cell, monoclonal antibody, pig

## Abstract

CLEC12A has been proposed as a suitable target for delivering antigen to dendritic cells (DCs) to enhance vaccine efficacy both in human and mouse. In this study, we have characterized the porcine homolog of CLEC12A (poCLEC12A). Using new monoclonal antibodies (mAb), raised against its ectodomain, poCLEC12A was found to be expressed on alveolar macrophages, blood conventional type 1 and type 2 DCs and plasmacytoid DCs, but not on monocytes, T cells, B cells or NK cells, in contrast to its human and murine homologs. Western blot analysis showed that in alveolar macrophages this receptor is expressed both as a monomer and a dimer. After binding to DCs, anti- poCLEC12A mAb was efficiently internalized. No significant changes were observed in TNFα or IFNα secretion by plasmacytoid DCs stimulated with either CpGs (ODN2216) or polyinosinic-polycytidylic acid (poly I:C), upon incubation with mAb. These results provide the basis for future investigations aimed to assess the ability of anti-poCLEC12A mAbs to improve vaccine efficacy by targeting antigen to DCs.

## Introduction

Innate immune cells express a diverse array of receptors, called pattern recognition receptors (PRRs), through which they sense the presence of invading microorganisms and monitor changes in tissue environment. These PRRs recognize microbial components, known as pathogen-associated molecular patterns as well as endogenous ligands released from stressed or damaged cells, referred as damage-associated molecular patterns (1).

One important group of PRRs is made of the myeloid C-type lectin receptors (CLRs), a large family of proteins defined by the presence of one or more C-type lectin-like domains (CTLD). Based on the arrangement of their CTLDs and phylogenetic considerations, CLRs have been classified into several groups (I-XVII) (2, 3). Myeloid CLRs have also been grouped depending on the signaling motifs present in their cytoplasmic tails or their association with adaptor molecules bearing signaling motifs (4, 5). Some CLRs contain an immunoreceptor tyrosine-based activation (ITAM)-like motif within their cytoplasmic tails (Dectin-1, CLEC-2 or DNGR-1), or associate with ITAM-bearing adaptor molecules (Dectin-2, CLECSF8 or Mincle). Activation of these receptors leads to intracellular signaling through spleen tyrosine kinase (Syk)-dependent pathways which result in the induction of nuclear factor-κB (NF-κB)-dependent pro-inflammatory responses (6, 7). Other CLRs, like CLEC-12A, possess an immunoreceptor tyrosine-based inhibition (ITIM)-motif in their cytoplasmic tails, which can recruit tyrosine phosphatases such as SHP-1 and SHP-2 upon receptor engagement. These phosphatases negatively regulate kinase-associated signaling pathways activated by heterologous receptors, thereby leading to the inhibition of cellular activation (8).

CLEC12A, also known as MICL or CD371, is a type II transmembrane protein classified within group V of CLRs. It has a single extracellular CTLD domain connected via a stalk region to a transmembrane segment and a short cytoplasmic tail containing an ITIM motif. In humans, CLEC12A is mainly expressed on myeloid cells, being found on monocytes, granulocytes, macrophages and DC subsets, and at lower levels on CD4+ T cells (9–11). In mouse, CLEC12A is expressed on DCs (CD8+ DCs and plasmacytoid DCs), monocytes, macrophages and granulocytes, but also on B cells and CD8+ T cells (12). No data are available on expression of porcine CLEC12A protein on different leukocyte subsets; however, transcriptomic analyses indicate the expression of mRNA coding for this molecule in DC subsets (13, 14).

The high expression of CLEC12A on various DC subsets, together with its endocytic capacity have led to investigate the potential of monoclonal antibodies (mAbs) against this molecule for delivering antigen to these cells as a way to improve vaccine efficacy and control the type of specific immune response elicited (10, 15, 16). In humans and mice, antigen targeted to CLEC12A has been shown to be efficiently processed by conventional DCs (cDCs) and plasmacytoid DCs (pDCs) and delivered to the MHC II and MHC I presentation pathways for activation of CD4 and CD8 T cells (15, 17).

In this study, we have characterized the porcine homolog of CLEC12A. Using mAbs raised against its extracellular region, poCLEC12A was found to be preferentially expressed on blood dendritic cells (pDCs and cDC subsets) and on alveolar macrophages. In the latter cells, it is expressed both as a monomer and a dimer. Antibody engagement of poCLEC12A leads to its internalization in all DC subsets. No significant effects were observed on the secretion of IFN-α or TNF-α by pDCs in response to CpGs or poly I:C, when these cells were incubated with anti-poCLEC12A mAb.

## Materials and Methods

### Animals and Cells

Blood samples were obtained from 6 to 12-month-old outbred Large-White pigs. PBMC and granulocytes were isolated on Percoll discontinuous gradients after blood sedimentation through dextran. When required, erythrocytes were lysed by incubation of cells in 0.15M NH_4_Cl, 10 mM NaHCO_3_, pH 7.4 for 5 min. Granulocytes were recovered from the lower Percoll phase, after lysis of residual erythrocytes by hypotonic treatment. Cells were resuspended in RPMI 1640 medium containing 10% FCS, 2 mM L-glutamine, 20 mM Hepes, 5 × 10^−5^ M 2-mercaptoethanol and 50 μg/ml gentamicin (complete medium).

Alveolar macrophages were collected by broncho-alveolar lavage of lungs obtained from healthy, conventionally reared, 3 to 6-week-old Large-White pigs that had been anesthetized with sodium pentobarbital (0.3 gr/Kg), and euthanized by exsanguination.

To generate monocyte-derived macrophages (moMØ), monocytes were isolated by magnetic sorting using anti-CD172a mAb BA1C11 and an AutoMACS cell sorter (Miltenyi Biotec, Bergisch-Gladbach, Germany), and cultured for 5 days in complete medium supplemented with 20 ng/ml of recombinant human (rh)M-CSF (Gibco Life Technologies, USA). Monocyte-derived dendritic cells (moDCs) were generated by culturing CD172a-sorted monocytes in medium supplemented with recombinant porcine (rp)GM-CSF (50 ng/ml) and rpIL-4 (50 ng/ml) (from Invitrogen, Carlsbad, CA, and Biosource Europe, Nivelles, Belgium, respectively) as previously described (18). On day 5, rpTNF-α (5 ng/ml, Biosource Europe) was added for 48 h to induce their maturation.

For confocal microscope analysis, pDCs were enriched by depleting PBMC suspensions with a cocktail of mAbs to CD3 (T cells), Siglec-10 (B cells), CD163 (monocytes) and CD11R3 (monocytes and cDCs) and goat anti-mouse IgG magnetic microbeads using a CS column and the VarioMACS cell sorting system (Miltenyi Biotec).

Chinese hamster ovary (CHO) cells were grown in Dulbecco's modified Eagle's minimal essential medium (DMEM) supplemented with 5% FCS, 2 mM L-glutamine and 50 μg/ml gentamicin.

All reported experiments have been executed in full compliance with guidelines by the ethical and animal welfare committees of the Institute (CEEA 2015-008 and PROEX 259/L5).

### Construction of Eukaryotic Expression Vectors for Porcine CLEC12A

The full-length cDNA of poCLEC12A (Clone: AMP01_009_C01, Accession number: AK230553.1) was isolated from a cDNA library of porcine alveolar macrophages as described previously (19). To produce a soluble form of this protein fused to the Fc fragment of human IgG1 (CLEC12A-Fc fusion protein), the sequence coding for the extracellular domain (ECD) of poCLEC12A was amplified by PCR using oligonucleotide primers (ECD C12F and ECD C12R) containing NheI and XbaI restriction sequences (Table 1). The PCR product was cloned in frame containing the coding sequence of the Fc portion of human IgG1 in a pcDNA 3.1 vector (pECD-Fc) (20). Finally, a sequence corresponding to CCL20 signal peptide (SP) was added by ligation after digestion, with NheI, of both SP and pECD-Fc (poCLEC12A-Fc).

**Table 1:** Oligonucleotide primers used in this work.

**Gen**	**Primer**	**Sequence**	**T_**AN**_ °C**	**bp**
CLEC12A-Nt-GFP	C12NtGFPF	ATG TCT GAA GAA GTC ACT TAT GCA GAT C	58	792/639
	C12NtGFPR	CTA CAT TCT TCC AGC TGG TTC TTC AC		
CLEC12A-Fc	ECD C12F	ATT AGC TAG C GA AAA GTT GAA TAA ACT ACA AAA TTT CAA TGA AGA ACT TC	5 c 58°/30 c 62°	614
	ECD C12R	ATA T TC TAG A CA TTC TTC CAG CAG GTT CTT CAC TTG TTA GTA TAT CC		
	SP F	ATT AGC TAG CCT TGA GCT GAA AAT GAT GTG CAG TAG C	5 c 56°/30 c 63°	122
	SP R	ATT AGC TAG CAT TGC TTG CTG CTT CTG ACT TGC		

The whole coding sequence of poCLEC12A was PCR amplified using oligonucleotide primers (C12NtGFPF and C12NtGFPR) shown in Table 1 and Biotools DNA polymerase (Biotools, Spain), and subcloned into pcDNA3.1/Nt-GFP-TOPO, following manufacturer protocol, to obtain CLEC12A fused to GFP at its N-terminal end.

Integrity, fidelity and orientation of cDNA sequences in selected clones were confirmed by sequencing.

### Expression of CLEC12A Constructs and Generation of Transfectants

CHO cells were transfected with plasmids poCLEC12A-Nt-GFP or poCLEC12A-Fc by using the LipofectAMINE PLUS reagent (Invitrogen, San Diego, CA, USA) accordingly to the manufacturer's instructions. Expression of GFP fusion protein was directly assessed by flow cytometry at 24 h post-transfection on a FACSCanto II cytometer (Becton Dickinson, San Jose, CA, USA). For analysis of expression of Fc fusion protein, monensin (2 μg/ml, Sigma) was added 4 h before harvesting. Cells were washed, permeabilized with cold methanol 10 min at −20°C, and incubated for 30 min at 4°C with FITC-conjugated goat anti-human IgG (Bio-Rad Laboratories, Hercules, CA, USA). After three washes, cells were analyzed by flow cytometry. Non-transfected cells were used as negative controls.

To obtain transfected cells stably expressing the poCLEC12A-Fc construct, after 1 day in culture transfected CHO cells were subjected to two rounds of limiting dilution cloning in presence of G-418 sulfate (Gibco Life Technologies), added at a concentration of 0.8 mg/ml in DMEM. Individual clones were examined for expression of the fusion protein by ELISA and/or flow cytometry, and Western analysis and expanded if positive. Positive clones that maintained expression were used in subsequent experiments and for the production of the fusion protein (Supplementary Figure 1).

### Purification of Recombinant Chimeric poCLEC12A-Fc Protein

PoCLEC12A-Fc was purified from culture supernatants of transfected CHO cells by affinity chromatography using a column of Sepharose 4 Fast Flow coupled with a llama antibody fragment recognizing human IgG (CaptureSelect human Fc, Thermofisher, Leiden, The Netherlands). Bound protein was eluted with glycine buffer (0.1 M, pH 2.7), followed by neutralization with 5% vol/vol Tris-HCl 1 M, pH 8.5. Proteins were dialyzed against PBS and the concentration estimated by absorbance at 280 nm and with a homemade ELISA for quantifying human IgG. PoCLEC12A-Fc purity was assessed by Coomassie Blue staining after SDS-PAGE.

### Monoclonal Antibody Production

Mice were immunized subcutaneously with 8 μg of recombinant poCLEC12A-Fc in Freund's complete adjuvant and boosted 2 and 4 weeks later with the same amount in Freund's incomplete adjuvant. Serum from immunized mice was collected 7–10 days after second boost, and the presence of specific Abs was tested by ELISA on plates coated with poCLEC12A-Fc and by flow cytometry on alveolar macrophages. Selected mice were boosted i.v. and i.p. with 8 μg of recombinant poCLEC12A-Fc by each route in 0.1 ml sterile PBS, and 4 days later spleen lymphocytes were fused with the SP2/0 murine plasmacytoma, using polyethylene glycol 4,000 (Merck, West Point, PA, USA) according to standard protocols (21).

Antibody production by hybridomas was screened by ELISA on plates coated with poCLEC12A-Fc and by flow cytometry on CHO transfectants expressing GFP-tagged poCLEC12A. Hybridomas secreting specific antibodies were cloned twice by limiting dilution. Class and subclass of mAbs were determined by ELISA with a mouse monoclonal antibody isotyping test kit from BioRad (Oxford, UK).

The characteristics of other mAbs used in this study are summarized in Table 2. When required, mAbs were purified by affinity chromatography using a Sepharose 4 Fast Flow resin coupled with an antibody recognizing mouse kappa L chains (CaptureSelect LC-kappa (mur), Thermofisher). MAbs were labeled with N-hydroxysuccinimidobiotin (Sigma) following standard protocols.

**Table 2:** List of mAbs used in this study.

**Specificity**	**Clone**	**Isotype**	**Source**
CD1	76-7-4	IgG2a	J. Lunney, ARS, USDA
CD3	BB23-8E6	IgG2b	M. Pescovitz, Indiana Univ. USA
CD4	74-12-4	IgG2b	J. Lunney, ARS, USDA
CD8α	76-2-11	IgG2a	J. Lunney, ARS, USDA
wCD11R1	MIL-4	IgG1	K. Haverson, Univ Bristol, UK
wCD11R3	2F4/11	IgG1	Own production, INIA
CD14	MIL-2	IgG2b	Bio-Rad
CD21	BB6-11C96	IgG1	Southern Biotech
CD163	2A10/11	IgG1	Own production, INIA
CD172a	BA1C11	IgG1	Own production, INIA
CD172a	74-22-15a	IgG2b	J. Lunney, ARS, USDA
CD203a	PM18-7	IgG1	AbD-Serotec
CADM1	3E1	IgY	MBL international
SLA-DR	1D2/CR4	IgG2a	Own production, INIA
Siglec 10	2E9	IgG1	Own production, INIA
GFP (HRP-conjugated)		IgG1	Miltenyi

### ELISA Epitope Mapping Assay

Polysorp microtiter plates (Nunc-Thermo Scientific, Roskilde, Denmark) were coated overnight at 4°C with 50 ng/well of purified poCLEC12A-Fc diluted in PBS. After blocking residual binding sites with PBS-1% bovine serum albumin (BSA) for 1 h at 37°C, plates were incubated for 60 min at room temperature (RT) with biotinylated antibodies (15 ng/well) mixed with increasing amounts of unlabeled purified mAbs, in dilution buffer (PBS, 0.1% BSA, 0.05% Tween 20). After five washes with tap water containing 0.1% Tween 20 (washing solution), the plates were incubated with horseradish peroxidase (HRP)-conjugated streptavidin (Thermo Fisher Scientific, Rockford, IL, USA) diluted to 1/2000 in dilution buffer, for 45 min at RT. Plates were washed five times, and the enzymatic reaction was visualized using OPD (Sigma) and measured on a Tecan GENios microplate reader at 492 nm. The results were expressed as percentages of inhibition using the following formula: (OD in absence of unlabeled mAb-OD in the presence of unlabeled mAb) × 100/OD in the absence of unlabeled mAb.

### Flow Cytometric Analysis and Cell Sorting

For single-color staining, cells (2–5 × 10^5^/well) were incubated with biotinylated mAbs to poCLEC12A for 30 min at 4°C. After two washes in PBS containing 0.1% BSA and 0.01% sodium azide (FACS buffer), cells were incubated with streptavidin-BV421 (BD Biosciences), for 30 min at 4°C. Then they were washed and fixed in 0.1% formaldehyde prior to analysis in a FACSCanto II flow cytometer. Biotin-labeled mouse IgG1 and IgG2a irrelevant mAbs were used as negative controls.

For two-color staining, PBMCs (2–5 × 10^5^/well) were first incubated with mAbs to CD3, CD4, CD8α, CD21 or CD172a, followed by staining with PE-conjugated rabbit F(ab')2 anti-mouse Igs (Dako). After blocking free binding sites of secondary antibodies with 10% normal mouse serum for 10 min, cells were incubated with biotin-labeled mAb to poCLEC12A followed by streptavidin-BV421. Isotype-matched irrelevant mouse IgG1, IgG2a and IgG2b, and biotin-labeled mouse IgG1 and IgG2a were used as negative controls.

For analysis of pDCs, PBMCs (10^6^/well) were first stained with anti-CD172a mAb BA1C11 (IgG1) and anti-CD4 mAb 74-12-4 (IgG2b) followed by APC-conjugated goat anti-mouse IgG1 and PE-conjugated goat anti-mouse IgG2b (both from Southern Biotech, Birmingham, AL, USA). After blocking free binding sites with 10% normal mouse serum, cells were incubated with biotinylated anti-CLEC12A mAb FA2B10 and streptavidin-BV421. Isotype-matched irrelevant mouse IgG1 and IgG2b and biotin-labeled mouse IgG1 were used as negative control antibodies.

For staining of blood cDCs, PBMCs (10^6^/well) were first incubated with anti-CADM1 (3E1, IgY), anti-CD172a (74-22-15, IgG2b) and biotin-labeled anti-CLEC12A (FA2B10) mAbs followed by PE-conjugated goat anti-mouse IgG2b and Alexa Fluor 647-conjugated goat anti-chicken IgY (Abcam, Cambridge, UK). After blocking free binding sites with normal mouse serum, cells were incubated with FITC-conjugated anti-CD14 (MIL-2) and streptavidin BV421. Isotype-matched irrelevant chicken IgY, mouse IgG2b, biotin-labeled mouse IgG1 and FITC-conjugated mouse IgG2b were used as negative control antibodies.

For sorting of pDCs for cytokine analysis, blood pDCs previously enriched by magnetic depletion as described above were incubated with mAbs to CD172a (BA1C11, IgG1) and CD4 (74-12-4, IgG2b) for 20 min at 4°C. After washing, cells were labeled with PE-conjugated goat anti-mouse IgG2b and APC-conjugated goat anti-mouse IgG1, for 20 min at 4°C. After a final wash, cells were suspended in PBS with 2 mM EDTA at 1 × 10^7^ cells/ml, filtered through a 70 μm nylon strainer (Thermofisher) for removal of cell clumps, and sorted using a FACSAria III cell sorter (Becton Dickinson). pDCs were gated as CD172a^+^ CD4^+^ cells and collected in RPMI 1640 medium containing 15% FCS. Non-specific binding was evaluated using isotype-matched control mAbs, and dead cells excluded with SYTOX blue dead cell stain (Life Technologies). Purity of the sorted population was higher than 90%.

### Internalization Assay

The internalization capacity of CLEC12A was analyzed by flow cytometry. PBMCs (10^6^) were incubated at 4°C for 30 min with 0.5 μg of biotinylated anti-poCLEC12A mAb FA2B10. After washing in FACS buffer without azide, cells were incubated at 37°C for different times (0–60 min) to allow endocytosis. Then, cells were labeled, at 4°C in presence of 0.01% sodium azide, with mAbs to CD172a and CADM1 followed by PE-conjugated goat anti-mouse IgG2b and Alexa Fluor 647-conjugated goat anti-chicken IgY. After blocking free binding sites with normal mouse serum, cells were incubated with FITC-conjugated anti-CD14 mAb MIL2, and streptavidin BV421. DCs were analyzed on a flow cytometer and gated as CD14^−^ CD172a^lo^ CADM1^+^ (cDC1) or CD14^−^ CD172a^hi^ CADM1^+^ (cDC2). To assess the internalization capacity in pDCs, after incubation with biotinylated anti-poCLEC12A mAb FA2B10, cells were labeled with anti-CD4 mAb and PE-conjugated goat anti-mouse IgG2b, following the assay as described above. CLEC12A internalization was calculated relative to the surface expression, based on the mean fluorescence intensity, of cells incubated 1 h at 4°C in the presence of 0.01% azide.

For confocal microscope analysis of CLEC12A internalization, pDCs were enriched from PBMC by magnetic depletion of CD3^+^, Siglec-10^+^, CD163^+^ and CD11R3^+^ cells by using the VarioMACS cell sorting system. The negative fraction was then incubated with biotin-labeled anti-CLEC12A mAb FA2B10 (1 μg/5 × 10^5^ cells) for 30 min on ice, followed by labeling with Alexa Fluor 488-conjugated streptavidin (Life Technologies) for 30 min on ice. Then, cells were incubated at 37°C for 60 min to allow endocytosis. Next, cells were surface stained with anti-CD4 mAb 74-12-4 (IgG2b) at 4°C followed by Alexa Fluor 594-conjugated goat anti-mouse IgG2b (Invitrogen) to identify pDCs. Thereafter, cells were fixed in PBS containing 0.1% formaldehyde for 20 min, settled on slides, and mounted in Prolong Gold (Invitrogen). Images were acquired with a Leica confocal laser scanning microscope SP8. Isotype controls did not show fluorescent staining. Pictures were analyzed with Image J.

### Western Blot Analyses

PoCLEC12A-Fc-transfected CHO cells (3 × 10^6^) were washed twice with PBS and solubilized in 0.3 ml of lysis buffer (50 mM Tris-HCl pH 7.4, 150 mM NaCl, 5 mM EDTA, 1% NP40, 10 μg/ml aprotinin and 1 mM phenylmethylsulfonylfluoride) for 1 h at 4°C. After centrifugation at 12,000 × g for 30 min, lysate supernatants were mixed with electrophoresis sample buffer (final concentration: 0.062 M Tris-HCl pH 6.8, 2% SDS, 10% glycerol, 0.001% bromophenol blue, with 0.7M 2-mercaptoethanol), boiled and run on a 10% SDS-PAGE. Separated proteins were transferred to nitrocellulose and analyzed by Western blotting with a biotin-conjugated goat anti-human IgG (eBiosciences) and streptavidin-HRP (Thermo Fisher Scientific). Peroxidase activity was visualized with the ECL detection assay following the recommendations of the manufacturer (GE Healthcare, Bio-Sciences AB).

Lysates of CHO cells expressing poCLEC12A-Nt-GFP, obtained as described in preceding paragraph, were resolved on a 10% SDS-PAGE, transferred to nitrocellulose and analyzed by Western blotting with an anti-GFP mAb conjugated to HRP (Miltenyi Biotec). Peroxidase activity was visualized with the ECL detection assay as described above.

Alveolar macrophages (10^8^) were washed twice with PBS and solubilized in 0.2 ml of lysis buffer for 1 h at 4°C. After centrifugation at 12,000 g for 30 min, the supernatant was mixed with the sample buffer and run on a 7.5% SDS-PAGE under reducing and non-reducing conditions. The separated proteins were transferred to nitrocellulose. Free binding sites on nitrocellulose were blocked with PBS 5% powdered milk. Thereafter, strips were incubated with hybridoma supernatants overnight at 4°C, followed by a 2 h incubation at RT with a peroxidase-labeled goat anti-mouse Ig (Dako), diluted in PBS, 2% powdered milk, 0.2% Tween 20. Peroxidase activity was visualized using the ECL detection assay.

### Cytokine Production

FACS-sorted pDCs were seeded, in triplicate, in round-bottom 96-well plates at 4 × 10^4^ cells/well in a final volume of 200 μl of complete medium alone or containing 0.5 μg of anti-CLEC12A mAb FA2B10 or an isotype matched control mAb. Cells were stimulated with either 5 μg/ml CpG/ODN2216 (InvivoGen, Toulouse, France), 10 μg/ml poly I:C (Sigma) or left unstimulated. After 18 h, supernatants were collected, and stored frozen at −80°C until assessment of cytokine production. Supernatants were analyzed for TNF-α content by using a commercial ELISA kit from Invitrogen following manufacturer's instructions. ELISA for IFN-α was performed using mAbs K9 and F17 from PBL Interferon Source (Piscataway, NJ), as previously described (22).

### Statistical Analysis

Statistical analyses of data were performed with the Mann–Whitney rank test, using GraphPad Prism 5.00 software (La Jolla, CA, USA). *P* < 0.05 was considered statistically significant.

## Results

### Structural Features of Porcine CLEC12A

Analysis of the 1,235 bp cDNA sequence contained in clone AK230553.1 showed that it encompassed a 792 bp open reading frame coding for a 263 amino-acid type II transmembrane protein. When the sequence of this protein was compared with that of CLEC12A homologs in mammalian species, available in databases, the amino acid identity ranged between 50% (mouse) and 68% (minke whale).

The C-terminal region of poCLEC12A contained one CTLD domain followed by a stalk segment. A hydrophobic sequence between positions 43 and 63 comprised the transmembrane region, as predicted by OCTOPUS program (23), after which was a cytoplasmic tail of 42 amino acids (Supplementary Figure 2). The CTLD domain, as predicted by InterPro program (24), contains six cysteines which are conserved in the species analyzed (human, mouse, cattle, horse, sheep, dog, cat, sperm whale, and minke whale). Out of them, four are likely involved in maintenance of the typical double loop-structure of these domains through the formation of two intrachain disulfide bonds (154–241; 220–233), whereas the other two cysteines, at the beginning of the CTLD sequence (126 and 137), would form other bond contributing to stabilize a β-hairpin at the base of the domain (2). The stalk segment contains two additional cysteines that may mediate homo- or heterodimerization of CLEC12A by forming interchain disulfide bonds. Up to five putative N glycosylation sites can also be identified in the sequence (3 in CTLD, 2 in stalk segment). Three of them, located at positions 81, 98, and 158, are conserved in human, mouse, cattle, horse, sheep, dog, cat, sperm whale, and minke whale, suggesting an important structural role. The cytoplasmic tail contains a tyrosine residue (Tyr-7) embedded in a segment (VTYADL) conserved in most of these species and which conforms well to the consensus ITIM motif for SH2 domain binding.

Analysis of the genomic sequence in database (accession number: NC_010447) revealed that *CLEC12A* gene is located at porcine chromosome 5 in a region that clusters several CLEC genes (1A, 1B, 2B, 7A, 9A, 12B). Po*CLEC12A* gene has 6 exons, with a genomic structure similar to that of human *CLEC12A*.

### CLEC12A Expression on Porcine Leukocyte Populations

**Figure 1:**
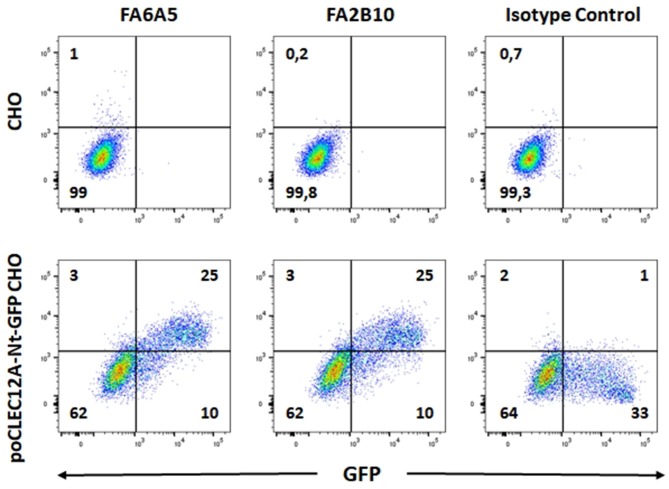
Reactivity of mAbs FA6A5 and FA2B10 with poCLEC12A transfectants. CHO cells transiently transfected with poCLEC12A-Nt-GFP construct were stained with mAbs FA6A5, FA2B10, or an IgG1 control antibody followed by APC-goat F(ab')2 anti-mouse Igs and analyzed by flow cytometry. A control staining of non-transfected CHO cells is also shown.

To examine the expression and biochemical properties of poCLEC12A, we generated mouse mAbs against the extracellular region of this receptor, using a soluble poCLEC12A-Fc fusion protein as immunogen. Two mAbs, FA2B10 and FA6A5, of IgG1 and IgG2a isotype, respectively, which bound to poCLEC12A-Fc fusion protein but not to human IgG in ELISA, were selected for further analysis. These mAbs did not recognize other Fc fusion proteins as tested with poCLEC12B-Fc or poCLEC4A-Fc (data not shown). Cross-blocking experiments using ELISA showed that none of these mAbs blocked binding of the other, suggesting that they bind to different epitopes on poCLEC12A molecule (Supplementary Figure 3). The specificity of both mAbs for poCLEC12A was further confirmed in flow cytometric analysis with CHO cells expressing the poCLEC12A-Nt-GFP construct (Figure 1).

**Figure 2:**
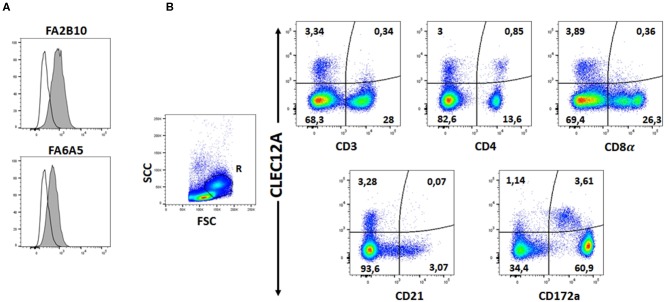
Expression of CLEC12A on porcine leukocyte subsets. **(A)** Flow cytometric analysis of porcine alveolar macrophages stained with biotin-conjugated mAb FA2B10 or FA6A5 followed by streptavidin BV421. Open histograms correspond to the negative control using an irrelevant isotype-matched mAb. **(B)** PBMC were double stained with anti-CD3, anti-CD4, anti-CD8α, anti-CD21 or anti-CD172a mAbs, and PE-conjugated rabbit F(ab')2 anti-mouse Ig (x-axis), followed by biotin-conjugated FA2B10 and streptavidin BV421 (y-axis). Isotype-matched irrelevant mAbs, unlabeled or biotin-labeled were used as negative controls. For analysis, cells were gated as FSC^hi^ (region R) to enrich the cDC population. Numbers indicate the percentage of cells within the respective quadrants. Results are representative of three independent experiments.

**Figure 3:**
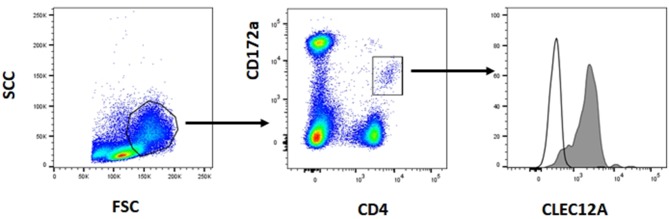
Expression of CLEC12A on plasmacytoid dendritic cells (pDCs). PBMC were first stained with anti-CD172a mAb BA1C11 and anti-CD4 mAb 74-12-4 followed by APC-conjugated goat anti-mouse IgG1 and PE-conjugated goat anti-mouse IgG2b. After blocking free binding sites, cells were incubated with biotin-labeled anti-CLEC12A mAb FA2B10 and streptavidin BV421. After doublet exclusion, cells with high FSC and SSC were selected and pDCs were identified as the CD172a^lo^ CD4^hi^ subpopulation. CLEC12A expression in gated population is shown in the filled histogram; open histogram corresponds to the negative control with an irrelevant isotype-matched mAb. The profiles shown are from a representative experiment out of three performed with cells from different donors.

**Figure 4:**
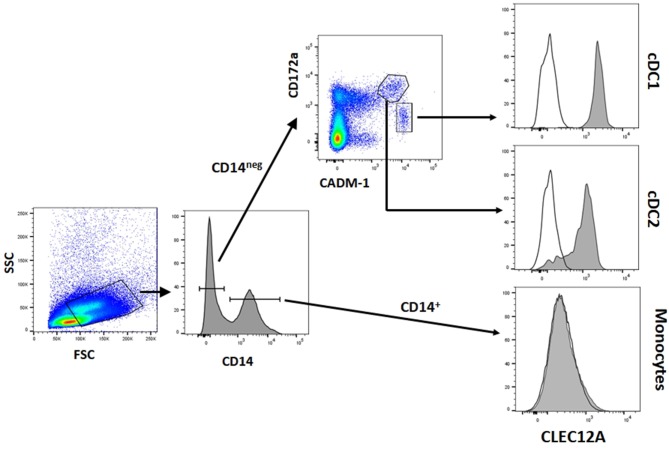
CLEC12A expression on blood conventional dendritic cells (cDCs). PBMC were first incubated with anti-CADM1 (3E1), anti-CD172a (74-22-15) and biotin-labeled anti-CLEC12A (FA2B10) mAbs followed by PE-conjugated goat anti-mouse IgG2b and Alexa Fluor 647-conjugated goat anti-chicken IgY. After blocking free binding sites, cells were incubated with FITC-conjugated anti-CD14 mAb MIL-2 and streptavidin BV421. Cells with high FSC and SSC were selected. Among these cells, cDC1 were gated as CD14^−^ CD172a^lo^ CADM1^+^, cDC2 as CD14^−^ CD172a^hi^ CADM1^+^, and monocytes as CD14^+^. Expression of CLEC12A in gated populations is shown as filled histograms; open histograms correspond to negative controls using an irrelevant isotype-matched mAb. The profiles shown are from a representative experiment out of three performed with cells from different donors.

The reactivity of these mAbs on different porcine leukocyte populations was analyzed by flow cytometry. Both mAbs stained alveolar macrophages (Figure 2A). However, in two-color flow cytometric analysis, only mAb FA2B10 clearly bound to a small population of CD172a^int/lo^ cells within PBMCs, part of which expressed high levels of CD4, a feature of pDCs (Figure 2B). No binding was observed on monocytes, identified as CD172a^hi^ cells; T cells, defined by the expression of CD3; NK cells, which are comprised within the CD8α^+/lo^ population; or B cells, identified as CD21^+^ cells. A three-color staining analysis with mAbs to CD172a, CD4, and FA2B10 showed the expression of CLEC12A on CD172a^+^ CD4^hi^ pDCs (Figure 3). In addition, four-color staining with mAbs to CD14, CD172a, CADM1, and FA2B10, showed that cDC1 (CD14^−^ CD172a^lo^ CADM1^+^) expressed higher levels of CLEC12A than cDC2 (CD14^−^ CD172a^hi^ CADM1^+^) (Figure 4). The correct identification of these cDC subsets was corroborated by analysis of a series of markers such as CD1, wCD11R1 or CD163, that are differentially expressed on these subsets and monocytes (13) (Supplementary Figure 4). Overall, based on these results, the expression of poCLEC12A seems more restricted than that of its mouse and human homologs which are broadly expressed in myeloid cells, as well as in some lymphocyte subsets.

### Biochemical Characterization of poCLEC12A

**Figure 5:**
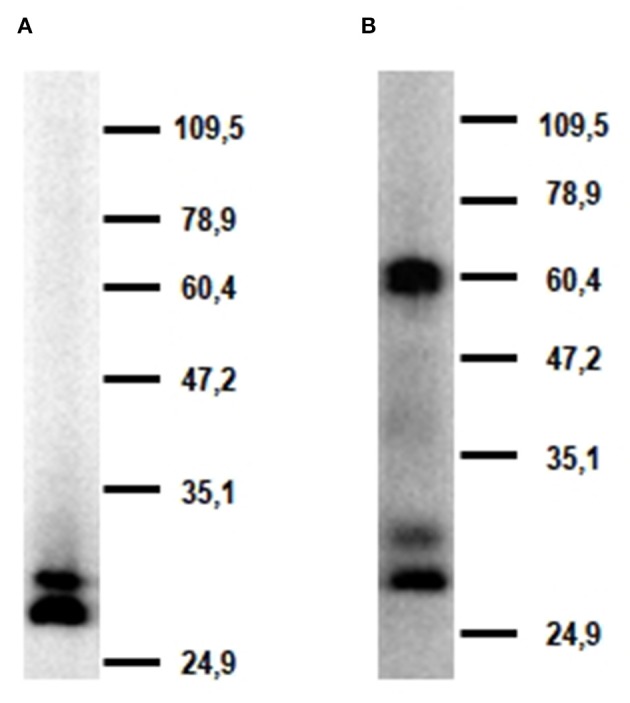
Molecular characterization of porcine CLEC12A. Lysates from alveolar macrophages were resolved by 7.5% SDS-PAGE under reducing **(A)** or non-reducing conditions **(B)**, transferred to nitrocellulose membranes and probed with mAb FA6A5. Numbers in the right indicate position and size of MW markers. Results are representative of two independent experiments.

Western blot analysis of alveolar macrophage lysates with mAb FA6A5 identified two very close bands with molecular masses around 30 kDa under reducing conditions (Figure 5A). These two bands were also detected under non-reducing conditions together with a higher band of approximately 60 kDa, indicating that porcine CLEC12A could be expressed in these cells both as a monomer and a dimer or higher molecular weight oligomer (Figure 5B). MAb FA2B10 failed to recognize any molecule under these conditions.

### Expression of CLEC12A on moMØ and moDCs

Since CLEC12A was expressed on alveolar macrophages but not on monocytes, we analyzed if CLEC12A expression could be induced when monocytes were differentiated into macrophages using rhM-CSF. No significant expression was detected even after 5 days of culture of monocytes with this cytokine, whereas the expression of CD203a, a marker associated with the differentiation of monocytes into macrophages (25), was clearly up-regulated at 72 h of culture. We neither detected CLEC12A expression on DCs derived from monocytes with GM-CSF and IL- 4, even after treatment, on day 5, with TNF-α to induce their maturation (Supplementary Figure 5).

### Effect of Anti-CLEC12A mAb FA2B10 on Cytokine Production

**Figure 6:**
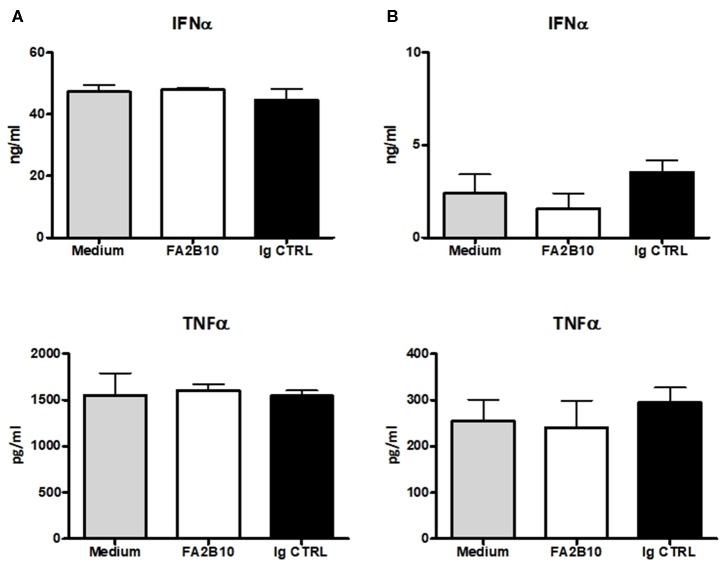
Effect of poCLEC12A engagement on the production of cytokines. FACS-sorted pDCs were cultured with anti-poCLEC12A mAb FA2B10, an isotype-matched control mAb or in medium alone, in the presence of either CpG/ODN2216 (5 μg/ml) **(A)** or poly I:C (10 μg/ml) **(B)** for 18 h, after which supernatants were collected and analyzed by ELISA for cytokine production. Results shown (mean + SD) are from a representative experiment, run in triplicates (number of performed experiments: 3 for CpG, 2 for poly I:C, performed with cells from different donors).

In humans, CLEC12A engagement with mAbs has been shown to lead to signals that modulate cytokine secretion by DCs (26). So we evaluated whether the addition of anti-CLEC12A mAb FA2B10 had an impact on the production of IFN-α or TNF-α by pDCs, which have been shown to produce high amounts of these cytokines in response to TLR3 or TLR9 stimulation (13, 14). For this purpose, pDCs were FACS-sorted and incubated with mAb FA2B10 or an isotype-matched control, in the presence or absence of CpG ODN2216 or poly I:C. After 18 h, the supernatants were harvested and the amount of secreted IFN-α or TNF-α determined by ELISA (Figure 6). No significant differences were detected in production of these cytokines in response to the stimuli tested, between cultures treated with mAb FA2B10 or an irrelevant isotype-matched control. No production of these cytokines was detected when cells were incubated with mAb FA2B10 alone.

### Internalization of CLEC12A Bound Antibody

**Figure 7:**
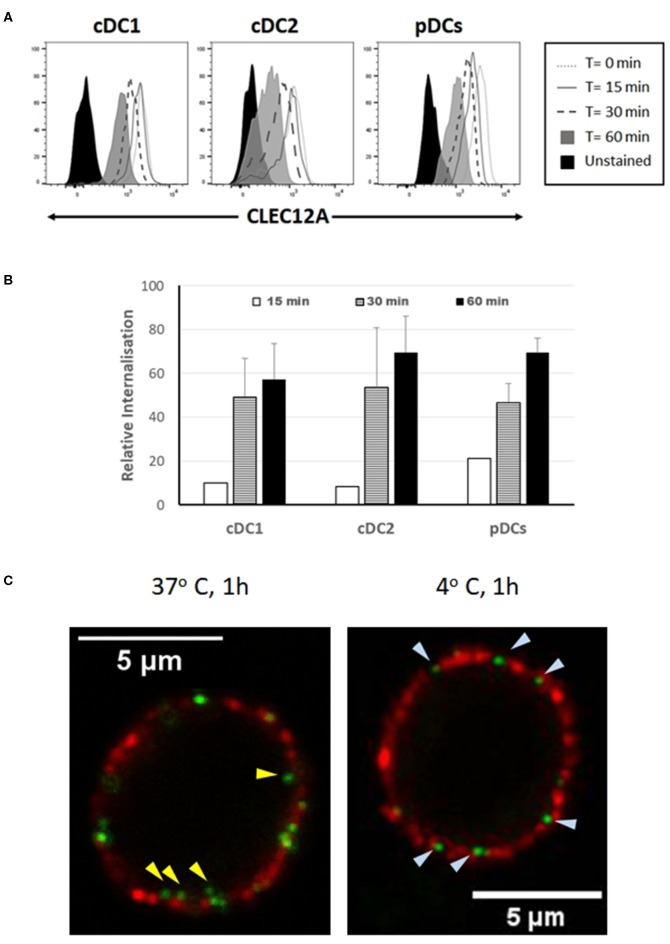
Binding of mAb FA2B10 to CLEC12A induces receptor internalization in blood DC subsets. **(A)** PBMCs were labeled with biotin-conjugated anti-CLEC12A mAb FA2B10, followed by 0- to 60-min incubation at 37°C. Surface expression of bound anti-CLEC12A mAb was analyzed by flow cytometry following labeling with streptavidin BV421. cDC subsets were identified according to the expression of CD14, CD172a, and CADM-1 markers; pDCs were identified based on the expression of CD4. Results are representative of three independent experiments. **(B)** CLEC12A internalization was calculated relative to the surface expression in cells kept at 4°C based on the mean fluorescence intensity. Data are shown as mean+SD of three independent experiments. **(C)** Analysis by confocal microscopy of CLEC12A internalization in pDCs. Cells were stained with biotinylated anti-CLEC12A mAb FA2B10, followed by labeling with Alexa Fluor 488-conjugated streptavidin (green). Then, cells were incubated at either 37°C, for allowing internalization, or 4°C, to prevent it, and subsequently stained with anti-CD4 mAb 74-12-4, and Alexa Fluor 594-conjugated anti-mouse IgG2b (red). Yellow arrowheads show anti-CLEC12A green labeled complexes inside cell; bluish arrowheads point to surface located anti-CLEC12A.

In humans and mice, CLEC12A engagement with mAb has been shown to induce the internalization of this receptor (10, 15). To investigate whether binding of mAb FA2B10 to poCLEC12A also led to its internalization, PBMCs were incubated with biotinylated mAb FA2B10 at 4°C, washed, and then warmed to 37°C for the indicated times to allow for receptor internalization. Afterwards, cells were stained with a combination of mAbs to CD14, CD172a, and CADM-1 followed by isotype-specific secondary antibodies to discriminate the different DC populations, and the amount of biotinylated anti–poCLEC12A mAb remaining on cell surface detected by using streptavidin-BV421. For pDC identification, cells were stained with an anti-CD4 mAb. A gradual reduction in poCLEC12A cell surface staining was evident after incubation at 37°C in both cDC1 and cDC2 subsets (Figure 7A). After 30 min of incubation, the mAb bound to the cell surface in cDC1 decreased >40%, compared with mean fluorescence intensity of cells maintained at 4°C, falling to ≈60% after 60 min. Comparable results were obtained for cDC2 and pDC, suggesting that after mAb engagement poCLEC12A was efficiently internalized by all DC subsets (Figure 7B).

CLEC12A internalization following cross-linking with mAb FA2B10 was also analyzed by confocal microscopy in pDCs enriched by magnetic depletion of CD3+, Siglec-10+, CD163+ and CD11R3+ cells, and identified by the expression of CD4 molecule. While in the cells incubated at 4°C CLEC12A was only observed in cell surface like CD4, in those incubated for 60 min at 37°C, clusters of CLEC12A were clearly detected inside the cells (Figure 7C).

## Discussion

In this study we describe the characterization of porcine homolog of CLEC12A using a cDNA clone isolated from an alveolar macrophage library, which encodes the full length CLEC12A isoform. Porcine CLEC12A displays 50–65% overall amino acid identity with its homologs in other mammal species being higher with cattle (64%), sheep (62%), horse (61%), dog (62%) or cat (64%), and lower with human (55%) or mouse (50%) (Table 3). Like its homologs in these species, porcine CLEC12A contains the six canonical cysteines in the CTLD and two extra cysteines in the stalk, which may allow covalent homo- or hetero-dimerization (9). Both a high 60 kDa and two lower molecular mass (around 30 kDa) bands were detected in western blot analysis with mAb FA6A5 on alveolar macrophage lysates under non-reducing conditions, which were resolved to two close bands of around 30 kDa under reducing conditions indicating that it can be expressed as a dimer or oligomer in these cells. The two bands around 30 kDa observed under reducing conditions could reflect differential glycosylation, other posttranscriptional modifications or the expression of splice variants of the receptor, as described in other species (9, 12, 27). Murine Clec12A has also been shown to be expressed as a dimer in contrast to human CLEC12A which was detected predominantly as a monomer. It has been argued that the more extensive glycosylation of human molecule might prevent its dimerization (12, 27). In this regard, it should be noted the stalk of poCLEC12A contains a lower number of potential N-glycosylation sites (2 vs. 4), when compared to human.

**Table 3:** Percent identity of porcine CLEC12A sequence to other CLEC12A sequences in data bases.

**Species (common name)**	**Sequence ID /accession number**	**Isoform**	**Aa % identity^(a)^**
*Homo sapiens* (man)	NP_612210.4	Isoform 1	56% (258)
*Mus musculus* (mouse)	NP_808354.1		50% (261)
*Bos Taurus* (cattle)	NP_001098815.1		64% (264)
*Ovis aries* (sheep)	XP_004006929.2	Isoform X1	62% (260)
*Equus caballus* (horse)	XP_001499515.2	Isoform X1	61% (285)
*Canis lupus familiaris* (dog)	XP_534891.2		62% (264)
*Felis catus* (cat)	XP_003988454.1	Isoform X1	64% (266)
*Balaenoptera acutorostrata* (minke whale)	XP_007196154.1	Isoform X1	68% (268)
*Physeter catodon* (sperm whale)	XP_028347294.1		61% (292)

*Sequence AK230553 used as query*.

(a) *In parenthesis length of compared sequences with optimum alignment*.

The distribution of poCLEC12A in different leukocyte populations was analyzed using mAbs raised against its ectodomain, as shown by their reactivity with CHO cell transfectants expressing a fusion protein of CLEC12A with GFP. Both mAbs developed in this work react with alveolar macrophages; however, only one, FA2B10, shows a clear binding to blood DCs. Based on the reactivity with this mAb, poCLEC12A appears to be preferentially expressed on plasmacytoid and conventional DCs in the blood, with a higher expression on cDC1 compared to cDC2. No expression was detected on monocytes, moDCs and moMØ. These results are in agreement with data from RNA-seq analysis reported by Auray et al. who also detected the highest levels of *CLEC12A* gene expression in porcine pDCs and cDC1, and lower in cDC2, whereas monocytes and moMØ were negative (13). Similar results were obtained by Edwards et al. in gene expression microarray analysis of porcine blood DC subsets sorted according to the expression of CD1 antigen, which found a higher expression of *CLEC12A* gene in CD1^−^ blood DCs, equivalent to the cDC1 population, compared to CD1^+^ DCs, which correspond to the cDC2 population (14). According to these data, the pattern of expression of poCLEC12A seems to be more restricted than that of the human and mouse homologs, which in addition to DCs are expressed on monocytes, moDCs, and some lymphocyte subsets (10, 12, 15, 26, 27). Nevertheless, in these species, pDC and cDC1 are also among the cells that express the highest levels of CLEC12A on their surface (10, 15).

At present we do not know the reasons that explain the different staining patterns of FA6A5 and FA2B10 mAbs. These mAbs recognize distinct non-overlapping epitopes as shown by cross-blocking experiments in ELISA, which could be differentially affected by the glycosylation or other post-translational modifications of the molecule. A significant variation in the level of N-glycosylation has been reported for human CLEC12A depending on the leukocyte populations analyzed (27). Another possible explanation for the different reactivity of mAbs would be the existence of splicing variants with a differential expression in distinct cell types. Splicing variants have been described for human CLEC12A (9) as well as in other members of the C-type lectin family, such as CLEC4A and dectin-1 (28, 29). In the latter, the two major isoforms have been shown to be differentially expressed in granulocytes and monocytes/macrophages (28). A third possibility is the formation of molecular complexes that prevent the access of antibody to the epitope. In fact, a single CLR might adopt different conformations in different cell types, as a result of its interactions with other CLRs, as has been shown for MCL (Clec4d) with Mincle (Clec4e) or Dectin2 (Clec4n) (30, 31).

Through the ITIM motif located in its cytoplasmic tail, CLEC12A can interact with SHP-1 and SHP-2 phosphatases and modulate cell functions (9, 12, 32). CLEC12A engagement has been shown to lead to different outcomes depending on the nature of the stimulus and the inflammatory context, and the antibody used in the assays. Hutten and colleagues reported that mAb binding to CLEC12A did not affect production of the immunoregulatory cytokine IL-10 or the pro-inflammatory cytokine TNF-α in human moDCs, cultured with various maturation stimuli (15). On the other hand, Chen and colleagues found that ligation of this receptor with mAbs suppressed the production of IL-12 and TNF-α induced by LPS in moDCs, whereas when the cells were stimulated with CD40L, it led to an increase in IL-12 and TNF-α production (26). In our study the binding of anti-poCLEC12A mAb FA2B10 alone does not result in production of IFN-α and TNF-α by pDCs and has not effects on the production of IFN-α or TNF-α upon stimulation of TLR3 or TLR9 with poly I:C or CpG (ODN2216), respectively.

A lack of effect on the activation of DCs after receptor engagement by this mAb may be beneficial regarding a potential use of it for targeting antigens to these APCs. Further studies will be required to examine potential immunomodulatory effects exerted *in vivo* by this mAb.

Like its human counterpart, poCLEC12A is capable of internalizing antigens for presentation to T cells. Both cDCs and pCDs efficiently internalized mAb FA2B10 bound to poCLEC12A. In humans, cDCs and pDCs have been shown to be able of cross presenting antigens delivered via CLEC12A, leading to activation of CD8^+^ T cells (15). Future studies will investigate if poCLEC12A is capable of shuttling antigens to the MHC class I cross-presentation pathway and elicit CD8 T-cell responses.

In summary, we have characterized the porcine homolog of CLEC12A and shown that it is predominantly expressed on blood DC populations. This finding together with preliminary results from functional assays provides the basis for future studies on the potential use of anti- CLEC12A mAb in vaccination strategies aimed to convey antigens to DCs in the pig. Besides, anti-CLEC12A mAb FA2B10 may be a very useful tool for studies on the biology of porcine DC subsets and their interaction with different pathogens, enabling an easy discrimination of peripheral blood DCs from monocytes and facilitating their isolation.

## Data Availability Statement

The datasets generated for this study are available on request to the corresponding author.

## Ethics Statement

The animal study was reviewed and approved by Comité ético de experimentación animal de INIA.

## Author Contributions

BÁ, AE, and JD designed the study. BÁ, EN-P, PM, TP, and CR performed the experiments. DT and HU provided the plasmids with the whole poCLEC12A coding sequence. AE supervised cloning and molecular issues. JD supervised mAb development and phenotyping and functional issues. BÁ, AE, and JD wrote the manuscript. All authors have seen and finally approved the version submitted for publication.

## Conflict of Interest

The authors declare that the research was conducted in the absence of any commercial or financial relationships that could be construed as a potential conflict of interest.
