# Supplementary material for: Characterization of the Porcine CLEC12A and Analysis of Its Expression on Blood Dendritic Cell Subsets
Source: Front Immunol. 2020 May 13;11:863. doi: 10.3389/fimmu.2020.00863 (PMC7237735; doi:10.3389/fimmu.2020.00863)
Supplement: Supplementary file 1 [file Data_Sheet_1.docx]

**
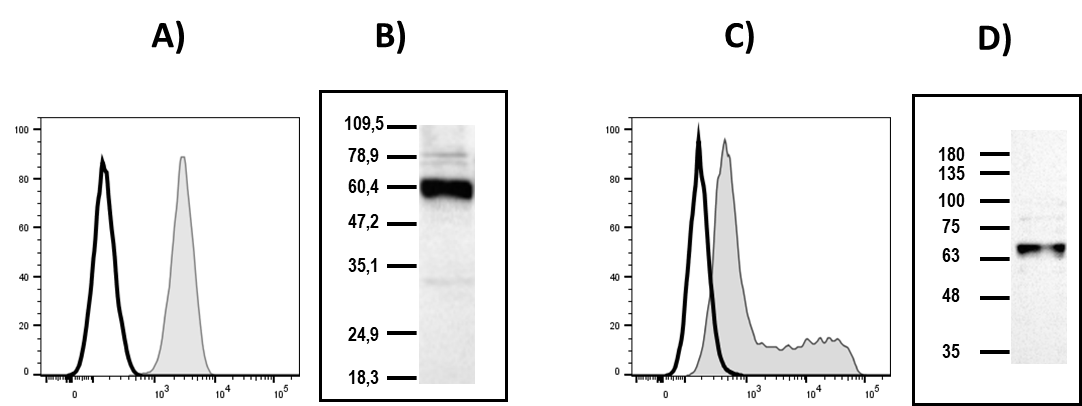
**

**Supplementary figure 1.** **Expression of porcine CLEC12A in transfected cells.** Flow cytometric analysis showing expression of poCLEC12A fused to Fc fragment of human IgG1 (A) or GFP (C) in transfected CHO cells (filled histograms). Non transfected CHO cells (open histogram) were used as negative control. Cell lysates were resolved by 10% SDS–PAGE under reducing conditions and the expression of CLEC12A fusion proteins analyzed by Western blotting with specific antibodies against either human IgG (B) or GFP (D). Numbers on the left indicate the position and size of MW markers.


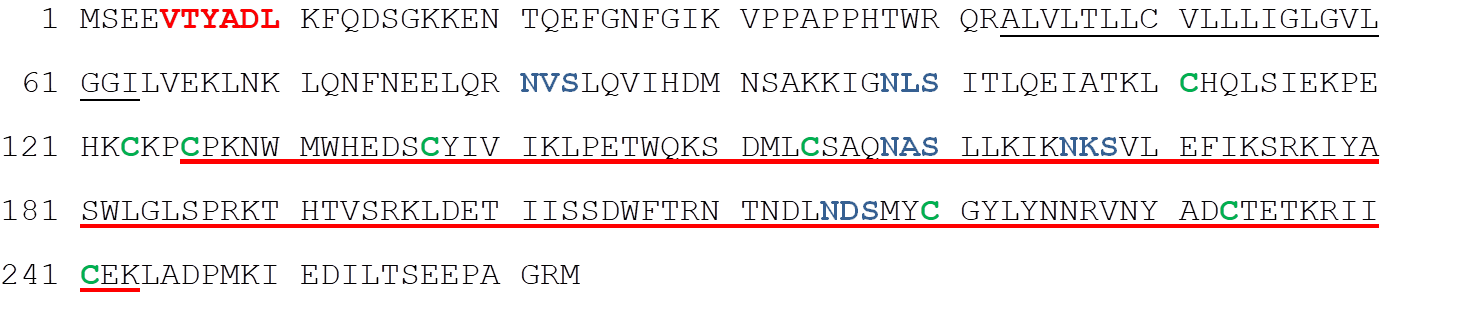


**Supplementary figure 2.** **Predicted amino-acid sequence of porcine CLEC12A protein used in this study** (Accession number: NM_001301669.1). The different segments of the protein are highlighted as follows: CTLD domain, underlined in red; transmembrane segment, underlined in black; ITIM motif, red. Cysteines are shown in green bold, and putative N-glycosylation sites in blue.


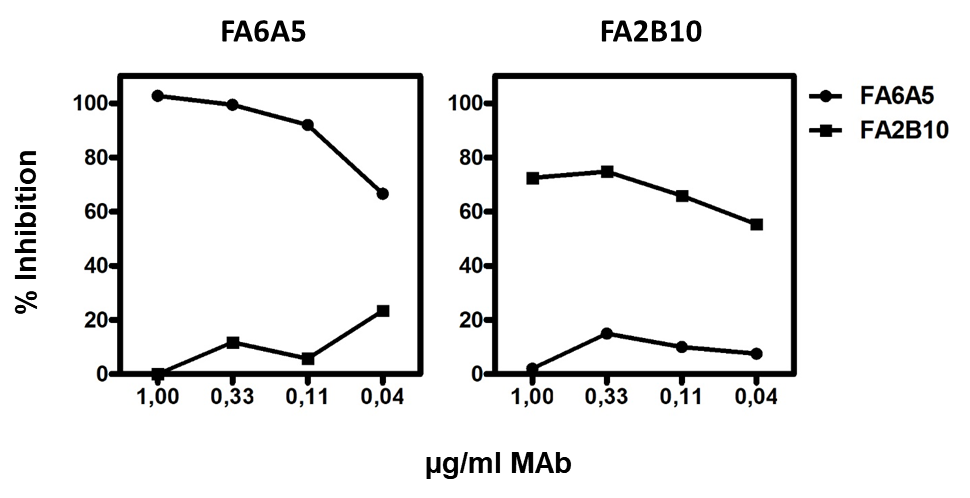


**Supplementary figure 3. Epitope mapping**. The binding of biotin-labeled mAb FA6A5 or FA2B10 to ELISA plates coated with recombinant protein CLEC12A-Fc was competed with different amounts of unlabeled FA2B10 ( **-◼-**) or FA6A5 ( **-⚫-**) mAbs. The results are expressed as % of inhibition, calculated as described in Materials and Methods.


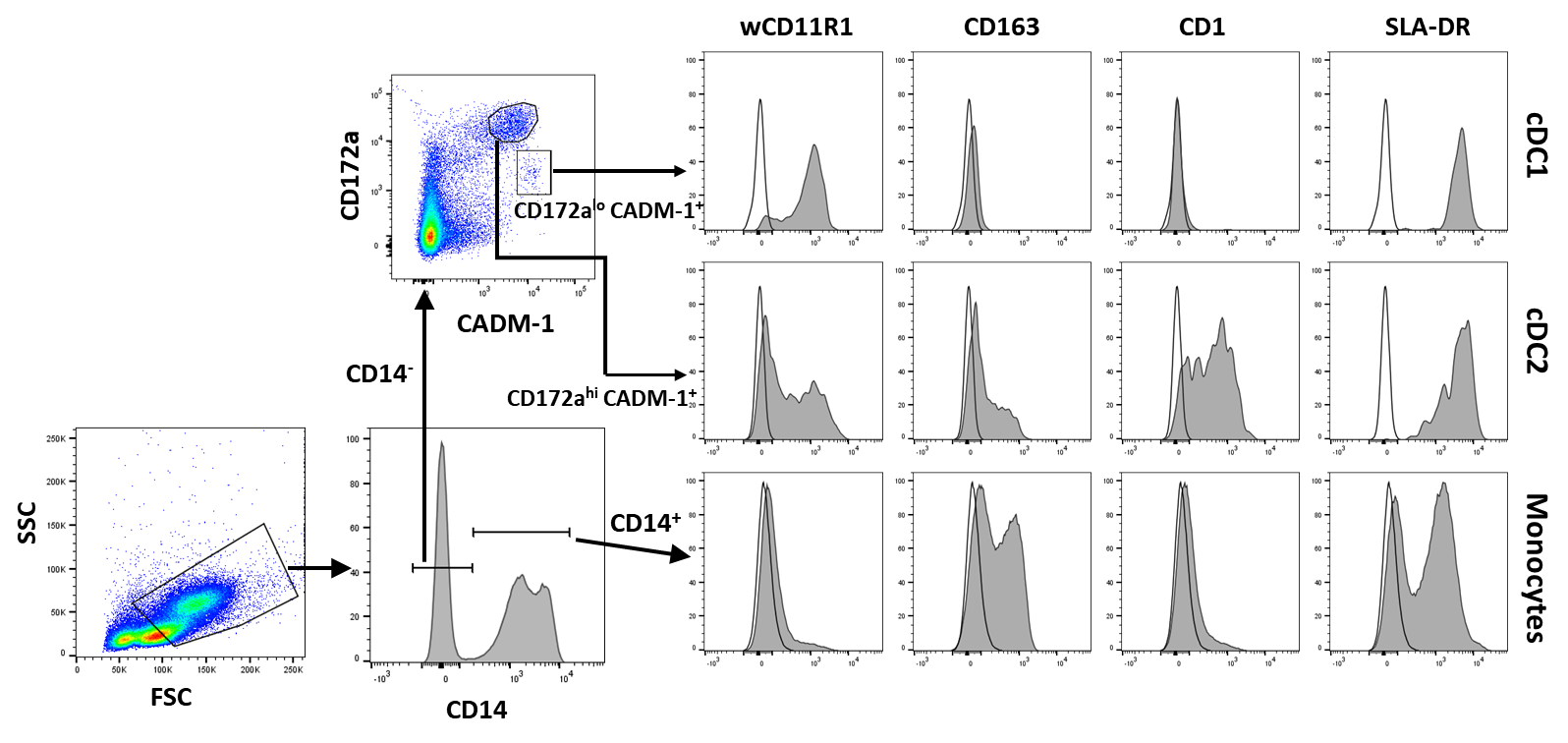


**Supplementary figure 4. Phenotype of cDC subsets.** PBMC were first incubated with anti-CD14 (MIL-2, IgG2b) and anti-CADM1 (3E1, IgY) and either anti-wCD11R1 (MIL-4, IgG1), anti-CD163 (2A10/11, IgG1), anti-CD1 (76-7-4, IgG2a) or anti-SLAII-DR (1D2/CR4, IgG2a) mAbs followed by APC-conjugated goat anti-mouse IgG1 or IgG2a, APC-Cy7-conjugated goat anti-mouse IgG2b and Alexa Fluor 488-conjugated goat anti-chicken IgY. After blocking free binding sites with 10% normal mouse serum, cells were incubated with biotin-labeled anti-CD172a mAb and streptavidin BV421. After doublet exclusion, cells with high FSC and SSC were selected. Among these cells, putative cDC1 were gated as CD14^-^ CD172a^lo^ CADM1^+^, cDC2 as CD14^-^ CD172a^hi^ CADM1^+^, and monocytes as CD14^+^. Expression of reference markers in gated populations is shown as filled histograms; open histograms correspond to negative controls using irrelevant isotype-matched mAbs. The profiles shown are from a representative experiment out of three performed with cells from different donors.


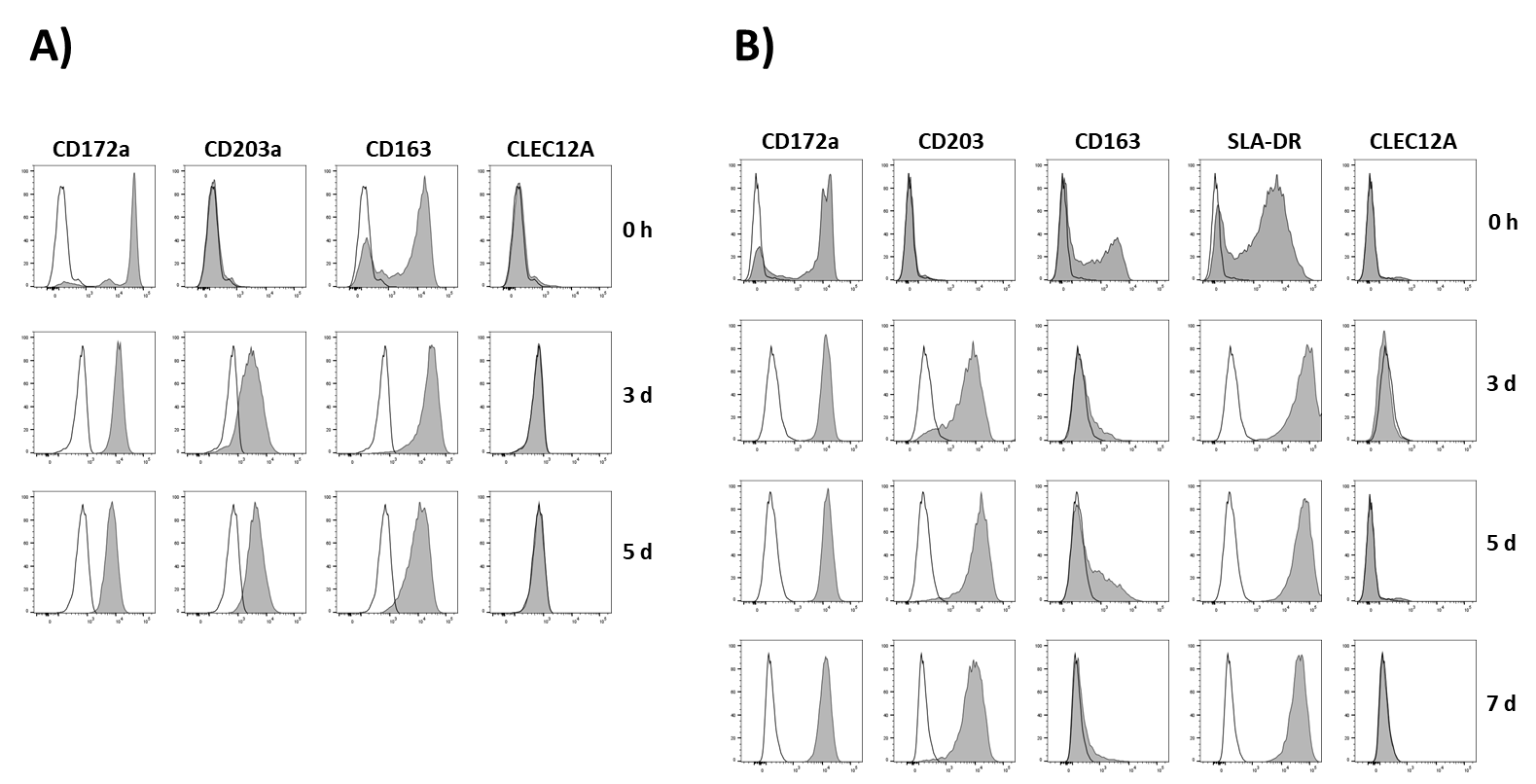


**Supplementary figure 5. Regulation of CLEC12A expression by cytokines.** A) Monocytes were sorted and cultured for 5 days in medium supplemented with rhM-CSF. At different times cells were harvested and the surface expression of CLEC12A and other reference markers analyzed by flow cytometry. B) Monocytes were differentiated into moDCs by culture for 7 days in medium supplemented with rpGM-CSF and rpIL-4, adding TNF-α on day 5 to induce maturation of cells. Cells were harvested and analyzed by flow cytometry on day 3, 5 and 7. Open histograms corresponds to the negative control staining using isotype-matched irrelevant mAbs. Results are representative of two independent experiments.
